# Bridging knowledge gaps: impact of remedial classes on first-year medical students in biochemistry – a cross-sectional study

**DOI:** 10.1186/s12909-024-06243-y

**Published:** 2024-11-26

**Authors:** Shreelaxmi V. Hegde, Prajna P. Shetty, Monikaa Senthilkumar, Ramesh Kandimalla, Grisilda Vidya Bernhardt, Janita R.T. Pinto, Renukadevi Mahadevan, Shashidhar M. Kotian, K.S. Rashmi

**Affiliations:** 1 Department of Biochemistry, Srinivas Institute of Medical Science and Research Centre, Mukka, Mangalore-574146, Rajiv Gandhi University of Health Sciences, Bangalore, Karnataka 560041 India; 2 Department of Biochemistry, Government Medical College, Narsampet-Warangal District, Telangana India; 3 Department of Biochemistry, RAKCOMS, RAK Medical and Health Sciences University, RAK, UAE; 4 Department of Biomedical Sciences, College of Medicine, Gulf Medical University, Ajman, UAE; 5 Department of Physiotherapy, College of Health Sciences, Gulf Medical University, Ajman, UAE; 6 Department of Research, Srinivas Institute of Medical Science and Research Centre, Srinivas University, Mangalore, Karnataka India; 7 Department of Physiology, Kasturba Medical College Mangalore, Manipal Academy of Higher Education, Manipal, Karnataka 576104 India

**Keywords:** Biochemistry, Feedback, Formative assessment, Medical undergraduates, Remediation, Summative assessment, Underperformers

## Abstract

**Background:**

Remedial teaching is a tailored educational approach dedicated to enhancing the academic performance of students facing challenges within the curriculum. By identifying and addressing specific learning difficulties, it provides essential support and guidance to bring students closer to expected standards while preventing future setbacks. We hypothesize that underperforming medical students who receive daily, tailored remediation will demonstrate significant improvement in their formative and summative assessment scores in biochemistry.

**Methods:**

A cross-sectional mixed-method study was conducted on 56 underperforming first-year medical undergraduates to assess the effect of targeted remediation on formative and summative assessments in Biochemistry. Training sessions included various remediation techniques over six months. Post-remediation feedback was collected to gather insights into students’ attitudes, perceptions, and the effectiveness of the methods in improving their understanding of the subject. Logistic regression analysis was employed to determine the most effective remediation for student performance. Benefits and weaknesses of remedial training approaches for future application as perceived by the students were derived through deductive thematic analysis of their feedback.

**Results:**

The mean marks, evaluated out of a maximum of 100, showed improvement from 29.86 ± 7.71 to 71.48 ± 10.19, with statistical significance (*p* < 0.001). From the students’ perspective, the most effective remediation method was grade incentives in formative assessments (odds ratio 6.19). Five major themes were identified: perceived barriers prior to remediation, positive outcomes and behavioral changes observed after remediation, and strengths and areas for improvement in remediation.

**Conclusions:**

The study concludes that identifying underperformers in the early stages of the medical curriculum and providing them with tailored remediation can enhance their performance in exams. Grade incentives in formative assessments, mind maps, quizzes, quick revisions, and assignments were beneficial remedial tools. Targeted remediation proved advantageous for students in improving their academic skills, exam preparation, time management, and attitudes towards the subject.

**Supplementary Information:**

The online version contains supplementary material available at 10.1186/s12909-024-06243-y.

## Background

The implementation of Competency-Based Medical Education (CBME) in India marks a transformative shift from traditional, teacher-centered approaches to a learner-centric paradigm. This innovative educational framework prioritizes the mastery of specific competencies by learners, ensuring they acquire essential skills and knowledge [1].

The commencement of students’ journeys into the medical curriculum presents a multitude of challenges, stemming from diverse educational backgrounds, varied aspirations, levels of motivation, learning aptitudes, and coping mechanisms for stress [2]. Central to addressing these challenges is the assessment of students’ academic performance, which serves as a pivotal tool for understanding and shaping the learning process. Timely intervention is crucial, particularly for students facing difficulties, as it enhances their chances of success and mitigates the risk of attrition from the course [3]. Formative assessment (FA), integrated seamlessly throughout the learning modules, plays a pivotal role in this regard [4]. By providing ongoing feedback and opportunities for self-assessment, FA enables learners to identify areas for improvement and actively engage in their own learning journey. Complementing this, summative assessment (SA), typically conducted at the conclusion of the academic year, serves as a comprehensive evaluation of the knowledge and skills acquired by students, informing decision-making processes and ensuring the maintenance of academic standards. Thus, a balanced approach to assessment, combining formative and summative methods, fosters an environment conducive to student growth, development, and retention within the medical curriculum [5].

However, not all medical students experience a smooth journey. In such situations, remediation is a crucial training strategy provided to underperforming medical students. Remedial intervention bridges gaps in medical knowledge, communication skills, and addresses attitudinal and motivational barriers encountered by students. Offering remediation early in the course has substantially led to positive outcomes. Lending credence to the effectiveness of remedial classes, in a study conducted by Joshi et al., 75 students underwent three-month targeted remedial training, significantly increasing the pass percentage in the second internal assessment compared to the first in anatomy [2]. Similarly in a study by Kumari et al., modified FA was delivered to 50 underperformers in biochemistry, resulting in a significant improvement in FA marks compared to controls [6]. The findings of the study by Murugesan et al., demonstrated a significant improvement in formative and summative assessment outcomes in pathology, post-remediation, when compared with the control group [7]. Stegers-Jager et al., conducted a short, integrated study skills programme (SSP) for first-year, poor-performing medical students. The effect of the SSP was measured by one of the following criteria: passing the first exam after the intervention, securing sufficient credits to proceed to the second year, and completing the first academic year within two attempts. This intervention benefited only a few students, with no significant improvement observed among the rest [8]. A systematic review on remediation for medical students described interventions ranging from 1 week to 1 year, with no single intervention strategy prevailing. The majority of studies used the format of the Observed Structured Clinical Examination (OSCE) for struggling learners. The review concluded that for a successful remediation strategy, early identification and re-examination with feedback to learners are crucial [9]. Moon et al., implemented 4 weeks of deliberate practice as an effective remediation intervention for underperforming 4th-year medical students. The study focused on improving clinical skills through role play, repetitive practice with standardized patient encounters, and OSCE [10].

A definitive and unified approach to guiding remediation training for students is currently lacking. Hence, this study aims to assess the efficacy of incorporating remedial training alongside standardized assessment tools, as recommended by the CBME curriculum, on both formative and summative outcomes among undergraduate medical students in biochemistry. The effectiveness of this remediation program has been evaluated through students’ feedback [11]. We hypothesize that implementing daily remedial training for underperforming medical students will lead to improvements in both FA and SA scores in biochemistry.

## Methods

### Study design

The study targeted underperforming first-year medical undergraduate students from the CBME 2022 batch in the Department of Biochemistry at Srinivas Institute of Medical Science and Research Centre, Mangalore, India. The batch comprised 150 students, aged 18 to 20 years, all taught according to the CBME curriculum. Sixty students who scored ≤ 40% in their second FA in theory were eligible for the remedial study. All eligible students participated in a one-hour orientation session with their mentors, where they were informed about the benefits of remediation and how the various activities would support their preparation for future assessments. Prior permission was obtained from the Institutional Research Committee, and informed consent was secured from the 56 participating students enrolled in the remedial class. A universal sampling strategy was used with a single-group pre-post study design. Upholding the principle of respecting individual autonomy, students unwilling to participate and students with aggregate marks > 40% were excluded from the study.

### Remedial training

The Biochemistry faculty, incorporating feedback from the 2021 CBME batch, designed an exclusive teaching methodology for the remedial students. The goal was to tailor the curriculum to meet the needs of underperforming students. Over six months, daily one-hour sessions included various teaching activities: mind maps, role play, viva, seminars, quizzes, small group discussions (SGD), weekly tests, quick revisions, assignments, grade incentives in FA, summarization, open-book tests, closed-book tests, poster making, underlining and highlighting points, leader-led group reading, independent study, and solving previous year’s papers, as detailed in Annexure 1. These sessions were in addition to the standard curriculum. All mentors underwent training for the structured remedial program before the study began. Every teaching activity employed in remedial intervention was based on students’ success in the evaluation process and their active participation. Therefore, few of these modules were continuously tailored based on the feedback and ongoing evaluation of students by their mentors.

### Questionnaire

A feedback questionnaire was distributed to students after the release of third FA results to mitigate response bias and ensure the quality of the remedial training. The questionnaire aimed to gather insights into students’ attitudes, perceptions of biochemistry, and the effectiveness of the remedial training in improving their understanding of the subject. It was meticulously developed by the first three authors through iterative surveys. Construct validity was established for the questionnaire, ensuring it accurately assessed the underlying theoretical concepts. To avoid subject bias, experts from other medical colleges who are active members of the medical education community reviewed and conducted the construct validation. Additionally, the questionnaire was pilot tested with third-year medical students to ensure acceptable reliability of the open-ended questions, using Cronbach’s α test.

The self-administered questionnaire comprised both closed and open-ended questions. Closed-ended questions used a five-point Likert scale (ranging from 1 to 5) to assess the effectiveness of various learning techniques. Open-ended questions solicited feedback on the strengths and weaknesses of the different remedial training methods employed. Participants were assured of the confidentiality of their responses, with the understanding that the study outcomes would be shared solely within the academic and scientific community.

### Compliance with remediation

Remedial students were monitored weekly, and periodic feedback on their progress was recorded. The rubric scoring method was used to assess students’ levels of achievement and participation in each remedial activity, as detailed in Table 1. A reward system, including added scores in progress reports based on periodic assessments and chocolates for active participation, incentivized student attendance, which was maintained by the department clerk.

**Table 1:** Grade criteria for remedial activities

Remediation	Score 5	Score 4	Score 3	Score 2	Score 1	Score 0
Viva/Quiz/ Weekly test	5 questions answered	4 questions answered	3 questions answered	2 questions answered	1 question answered	No answer
Assignments	Complete in all aspects with diagrams	NA	Good coverage	NA	Attempted	Not attended
Other modules	Attempted/ Attended	NA	NA	NA	NA	Not attempted/ Not attended

*Notes* The maximum score for all activities was 100. Each student’s score was then prorated to a scale of 6, and an equivalent grade incentive was added to their final score

### Statistical analysis

Statistical analysis was performed using SPSS software version 23.0, with a significance threshold set at *p* < 0.05. Descriptive statistics were utilized for data analysis. ANOVA with Bonferroni post-hoc tests were used for comparing internal marks across different time periods. Binary logistic regression analysis identified the most impactful remediation methods on student performance. Additionally, the Chi-square test was applied to examine the association between demographic factors and marks.

## Results

From the total 56 students selected, 51 underperformers successfully completed the remediation. 5 students dropped out of the remediation program for one of the following reasons – difficulty in coping with other subjects simultaneously, long distance-travel, did not want to stay after college hours, difficulty in adjusting with a different peer-group. The gender distribution among participants consisted of 32 males and 24 females, with 35 residing in hostel and 21 as day scholars. The mean marks in the first and second FAs were 24.44 ± 8.92 and 29.86 ± 7.71. The maximum marks for both the FA and SA were calculated out of 100. The third FA had a mean score of 68.14 ± 12.38, while the SA showed a mean score of 71.48 ± 10.19 (Table 2). There was a statistically significant improvement (*p* < 0.001) in third FA post remediation.

**Table 2:** Formative and summative marks of students

Assessment	Mean ± SD (*n* = 51)
First formative	24.44 ± 8.92
Second formative	29.86 ± 7.71
Third formative	68.14 ± 12.38
Summative	71.48 ± 10.19

*Notes* Data are presented as mean ± SD. SD stands for standard deviation. The marks are calculated out of a maximum of 100

A detailed inter-group comparison was conducted on the academic performance of remedial students across the assessment periods. The findings indicate a significant enhancement in educational achievement from the first and second FAs to the third and final SA. The data revealed substantial improvement in scores obtained by these students in the third FA, with no significant difference noted in comparison to the final SA (Table 3). Table 4 outlines the association between gender and type of accommodation (hostel or day scholar) with students’ overall performance in the SA. No statistically significant relationship was found between these demographic factors and the marks secured by students.

**Table 3:** Inter comparison of performance of students between different assessment periods

Assessment	Assessment	Mean difference	*p* value
First formative	Second formative	-5.41	0.036
	Third formative	-43.69	< 0.001*
	Summative	-47.04	< 0.001*
Second formative	Third formative	-38.28	< 0.001*
	Summative	-41.63	< 0.001*
Third formative	Summative	-3.34	0.510

*Notes* ‘*’ represents statistically significant values. Bonferroni post-hoc test was applied to get p values

**Table 4:** Association between performance and demographic characteristics in remediation

Demographics	Poor	Average	Good	Total number
Male	1	5	23	51 (*p* = 0.024)
Female	0	1	21	
Day scholar	0	0	17	51 (*p* = 0.132)
Hostelite	1	6	27	

*Notes* Student performance was graded into poor (< 40%), average (40–70%) and good (> 70%). x^2^ = 2.85 for gender; x^2^ = 4.057 for accommodation

To determine if these remedial resources were useful in overcoming their obstacles and achieving academic success, the frequency distribution of these separate domains was measured. Among 51 respondents, 70.6% and 21.5% graded the strategy of grade incentives in FA as very good and good, respectively. Nearly, 88.2% (very good 49.0% and good 39.2%), opined that conducting frequent quizzes was helpful. The majority of the participants that is 94% (very good 62.6% and good 31.4%) considered that quick revision of important topics had a positive impact. A greater number of remedial attendees, 96% (very good 76.4% and good 19.6%), supported role play as an interactive method to understand concepts which may help improve results. Nearly, 94.1% (23.5% very good, 41.2% good and acceptable 29.4%) graded mind map as a useful tool (Tables 5 and 6).

**Table 5:** Distribution of participants by their opinion about the usefulness of different remediation tools (grading 1 to 5)

Remediation	Likert scale	Frequency (*n* = 51)	Percentage (%)
Mind map	Very Poor	1	2.0
	Poor	2	3.9
	Acceptable	15	29.4
	Good	21	41.2
	Very Good	12	23.5
Role play	Very Poor	1	2.0
	Poor	0	0.0
	Acceptable	1	2.0
	Good	10	19.6
	Very Good	39	76.4
Viva	Very Poor	2	3.9
	Poor	1	2.0
	Acceptable	4	7.8
	Good	12	23.6
	Very Good	32	62.7
Seminar	Very Poor	3	6.0
	Poor	0	0.0
	Acceptable	8	16.0
	Good	22	44.0
	Very Good	18	34.0
Quiz	Very Poor	1	2.0
	Poor	0	0.0
	Acceptable	5	9.8
	Good	20	39.2
	Very Good	25	49.0

*Notes* Data are presented as n and %. Likert scale: 1 = Very poor to 5 = Very good

**Table 6:** Distribution of participants by their opinion about the usefulness of different remediation tools (grading 1 to 5)

Remediation	Likert scale	Frequency (*n* = 51)	Percentage (%)
SGD	Very Poor	1	2.0
	Poor	1	2.0
	Acceptable	11	21.5
	Good	17	33.3
	Very Good	21	41.2
Weekly test	Very Poor	1	2.0
	Poor	2	3.9
	Acceptable	4	7.8
	Good	17	33.4
	Very Good	27	52.9
Quick revision	Very Poor	1	2.0
	Poor	1	2.0
	Acceptable	1	2.0
	Good	16	31.4
	Very Good	32	62.6
Assignments	Very Poor	1	2.0
	Poor	12	23.5
	Acceptable	15	29.4
	Good	10	19.6
	Very Good	13	25.5
Grade incentives in FA	Very Poor	1	2.0
	Poor	0	0.0
	Acceptable	3	5.9
	Good	11	21.5
	Very Good	36	70.6

*Notes* Data are presented as n and %. Likert scale: 1 = Very poor to 5 = Very good

Furthermore, the frequency distribution of student’s views regarding usefulness of their involvement in less opted remedial methods revealed that open book test was more beneficial (88.3%) when compared to closed book test method (35.3%) (Table 7). Additionally, logistic regression analysis, was conducted to explore the effectiveness of various remedial methods (independent variables) in facilitating better performance (dependent variable) in the third FA (Table 8).

**Table 7:** Distribution of participants by their opinion about the usefulness of less frequently engaged remediation teaching methods (grading 1 to 5)

Remediation	Likert scale	Frequency (*n* = 51)	Percentage (%)
Summarization	Very Poor	0	0.0
	Poor	3	5.9
	Acceptable	11	21.6
	Good	31	60.8
	Very Good	6	11.7
Open-book test	Very Poor	0	0.0
	Poor	0	0.0
	Acceptable	6	11.7
	Good	44	86.3
	Very Good	1	2.0
Closed-book test	Very Poor	0	0.0
	Poor	1	2.0
	Acceptable	32	62.7
	Good	14	27.5
	Very Good	4	7.8
Poster making	Very Poor	0	0.0
	Poor	9	17.6
	Acceptable	26	51.0
	Good	14	27.5
	Very Good	2	3.9
Underlining and highlighting points	Very Poor	0	0.0
	Poor	2	3.9
	Acceptable	27	52.9
	Good	22	43.2
	Very Good	0	0.0
Leader-led group reading	Very Poor	0	0.0
	Poor	0	0.0
	Acceptable	29	56.9
	Good	22	43.1
	Very Good	0	0.0
Independent study	Very Poor	0	0.0
	Poor	29	56.9
	Acceptable	22	43.1
	Good	0	0.0
	Very Good	0	0.0
Solving previous year’s papers	Very Poor	0	0.0
	Poor	0	0.0
	Acceptable	29	56.9
	Good	22	43.1
	Very Good	0	0.0

*Notes* Data are presented as n and %. Likert scale: 1 = Very poor to 5 = Very good

**Table 8:** Logistic regression analysis showing the effect of different remediation tools on summative performance in students

Remediation tool	*p* value	Odds ratio	95% CI	
			Lower	Upper
Mind map	0.831	1.107	0.436	2.814
Role play	0.905	0.921	0.238	3.568
Seminar	0.089	0.396	0.136	1.153
Quiz	0.172	1.952	0.748	5.093
Viva	0.700	0.838	0.342	2.054
SGD	0.595	0.778	0.308	1.965
Weekly test	0.188	0.415	0.112	1.538
Quick revision	0.568	1.519	0.362	2.312
Assignments	0.789	1.106	0.528	2.320
Grade incentives in FA	0.025*	6.186	0.431	13.609

*Notes* CI is confidence interval. ‘*’ represents statistically significant values. Student performance was categorized into two groups: ‘good’ and ‘average.’ Based on their marks, 28 students (54.9%) were classified as ‘good,’ and 23 students (45.1%) were classified as ‘average’ in their SA. These performance groups served as the dependent variable in the binary logistic regression analysis, with various remediation methods as the independent variables

**Fig. 1:**
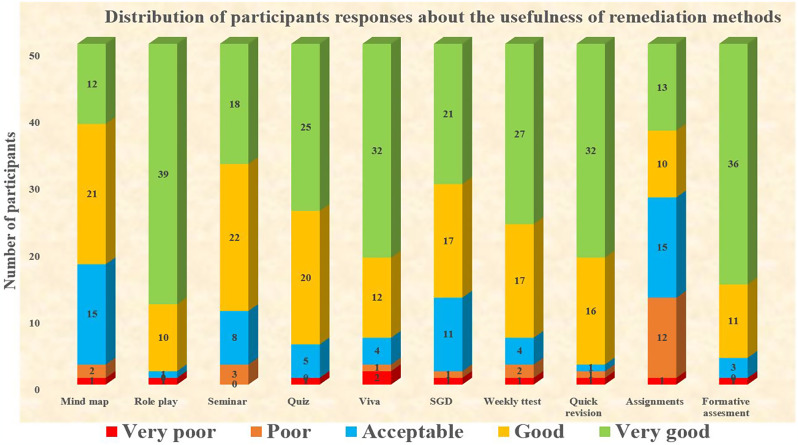
The distribution of participants’ responses about the usefulness of remediation methods is depicted in the figure. For mind map, 1 participant rated very poor, 2 as poor, 15 as acceptable, 21 as good, and 12 as very good. Role play received 1 very poor rating, 0 poor, 1 acceptable, 10 good, and 39 very good. Seminars were rated 3 very poor, 0 poor, 8 acceptable, 22 good, and 18 very good

The odds ratio of grade incentives in FA on overall performance of the students was found to be 6.19 (95% CI: 0.43–13.61), indicating a significant impact. Other remediation methods considered were quiz (odds ratio 1.95), quick revisions (odds ratio 1.52), mind map (odds ratio 1.11), and frequent assignments (odds ratio 1.11). Figure 1 illustrates the distribution of participants’ responses about the usefulness of remediation methods.

Utilizing a deductive approach, and analysing the effectiveness of various remedial methods several inductive themes and subthemes were identified from the students’ feedback.

### Theme:1 positive outcomes following remediation

Participants expressed an increased sense of confidence regarding the exam following the remediation process. This boost in confidence was attributed to the unwavering support provided by both the teaching and non-teaching staff of the department, who dedicated their work breaks and after-hours to actively participate in the remediation program.

Notably, 98% of participants credited their teachers’ unwavering dedication, continuous encouragement, and diligent efforts for their academic success. Additionally, 96% of students acknowledged that their teachers’ supportive attitude, recognition of efforts, expressions of gratitude, and rewards such as chocolates and improved FA scores significantly boosted their confidence. This encouragement facilitated active participation in remediation classes, enhanced comprehension of various biochemistry concepts, and instilled confidence for exams. (Data not in tables)

*The department’s consistent follow-up changed my approach and surprisingly led to improved attendance and a significant boost in my confidence.* (Participant 1, P-1)

The remediation study plan was meticulously designed to incorporate various techniques, such as role play, mind mapping, SGD, and weekly tests which facilitated repeated revision.

*I didn’t understand the theory classes at first*,* but after taking multiple tests and participating in group discussions*,* I now feel more confident about passing the final exam with a good grade.* (P2)

Around 66.7% of students acknowledged that their understanding of the topics and comprehension of the subject improved and enhanced through various training techniques of remediation. They initially perceived biochemistry as a challenging, tough, and confusing subject, but after remediation, they realized that it is more conceptual than erratic. (Data not in tables)

*At first*,* I found biochemistry very difficult. Now I feel I can easily read and score well in the subject.* (P3)

Nearly 86% and 92.2% of students believed that weekly tests and grade incentives during FAs, respectively had a positive impact on their skills, comprehension, and overall learning experience. (Data not in table)

*Because of daily basis questions on a particular chapter & daily tests during remedial made me like the subject.* (P4)

The study schedule was meticulously crafted to guarantee that the preparation for biochemistry was completed within a specific timeframe, fostering a sense of motivation among students to excel in the subject. Additionally, a positive learning environment was created, which included regular recognition and rewards for the students.

*Remedial hours enabled me to finish the biochemistry portion right away. I need not dedicate more time studying this subject.* (P5)

*Through remedial classes I could finish the topics I was lagging in*,* which enabled me to go through all the topics that have been covered in the respective weeks.* (P6)

*Teachers motivated us through various means*,* by giving chocolates*,* providing oral encouragement*,* and boosting grades in formative assessments. This motivated us to stay compliant to the remedial training.* (P7)

### Theme: 2 behavioural changes observed following remediation

Initially, students felt stigmatized to enrol in the remediation classes. They even wanted the remediation class to be renamed as “Extra Class,” and few eligible students wanted to remain out of this. A pre-session was engaged with the eligible candidates, and they were convinced of the benefits of the remediation classes. Mentors had to redefine the purpose of the remediation class and thereby remove the stigma around it.

*I hated chemistry since school days*,* mainly due to inorganic/organic part of it. I was not really into the subject. I always thought biochemistry was similar to chemistry. I’m grateful to be part of the remedial batch; it brought me closer to our teachers*,* who completely changed my view of boring biochemistry to a fun subject.* (P8)

*It was a nice experience. Initially*,* I was not interested in biochemistry*,* and I believed we could only catch up the day before the exam. However*,* I soon realized that wasn’t enough. My interest in biochemistry blossomed due to the department’s efforts. It was a good experience overall. Attending remedial classes gave me the time I needed to study biochemistry thoroughly and review the topics covered in the respective weeks.* (P9)

Optimum use of many of the study skills and remedial measures were also shared with the entire class from time to time.

*After the extra class*,* my hostel mates and I would gather in our room to discuss the chapters covered during the session. I even shared my highlighted notes with a friend who wasn’t part of the remedial class. Previously*,* we would discuss everything else other than biochemistry.* (P10)

Our study found that allowing students to showcase their talents, such as dancing, rapping, and presenting their opinions about the class, during the last 10–15 minutes of the remediation class had a positive impact on their involvement. Students enjoyed these activities and eagerly anticipated for more sessions. They made connections between the topics taught and their performances, fostering a sense of ownership and increasing their confidence. As a result, participants became more open, and their fear of being judged minimized. They believed that achieving a commendable score in the final evaluation would be a meaningful gift for their teachers.

*I got the opportunity twice to dance while my rapper buddy presented the topic of detoxification through his rapping method. Madam frequently rewarded us with chocolate.* (P11)

*I would like to see Ma’am happy. Her motivation motivates me to study. The only reason I even put efforts into Biochemistry is because of her. I knew that I would definitely pass in the end but without her I wouldn’t have found the subject interesting* (P12)

### Theme: 3 perceived barriers prior to remediation

Most of the students (86%) had perceived barriers towards biochemistry. Their responses revealed underlying subthemes, including a predisposition towards difficulty influenced by senior students and past experiences, a lack of structured study plans, and difficulties with reference textbooks. They viewed the subject as complex, confusing, and time-consuming. (Data not in tables)

*Studying biochemistry involves extensive revision for learning enzyme names*,* classifications*,* definitions*,* normal reference ranges*,* critical values*,* and Recommended Dietary Allowances (RDA)*,* remembering all of this was very difficult for us in the beginning.* (P13)

### Theme: 4 strengths of remediation

Remedial classes provided the students with a systematic approach to study. A well-structured study plan was created, and the students were required to comply with it. This proved to be particularly beneficial for those students who were struggling due to their prejudiced mindset against biochemistry.

*In the beginning*,* I liked the department but disliked the subject. Now*,* I love the department and the subject. The department and subject will definitely be my kingdom come for the finals and it has been an amazing journey.* (P14)

The implementation of diverse teaching methods to reinforce identical concepts was highly effective in enhancing students’ comprehension of key concepts. We discovered that individual mentorship between students and teachers fostered an environment where students felt comfortable expressing even their most insignificant doubts.

### Theme: 5 scope for improvement in remediation

When asked for recommendations to improve the design of the study plan, many students asked to start remediation programs as soon as the first formative assessment was completed and the poor performers are identified.

*Madam*,* I suggest that you arrange more & more test in the beginning itself for our juniors so that they will be familiar with each topic.* (P15)

The students proposed the elimination of the extensive assignments and seminars that were given at the start of the program, as they were considered unhelpful in understanding the concept. Students believed that shorter assignments given at the later phase of remediation which focused on important topics was much beneficial.

*I used to write assignments just to get them done*,* without really understanding what I was writing. Sometimes*,* I would even copy the content and then spend late nights studying biochemistry*,* which made me dislike the subject. However*,* when our teacher asked us to focus on selected questions*,* highlight key points*,* and take formative tests*,* it became much easier for us.* (P16)

*I think assignments could have been replaced*,* as they take a lot of time to write. It would be better if we received the assignment questions as tests instead.* (P17)

Post-remediation, 70% of students perceived all the methods as equally beneficial and did not feel the need for further improvement. However, a small subset of students (2%) who had actively participated in the remediation sessions still expressed worry about exams, which was later attributed to exam anxiety. This highlights the importance of addressing individual student needs and providing appropriate support mechanisms to mitigate exam-related stress. (Data not shown)

## Discussion

The study’s findings indicate that tailored remedial training for underperforming medical undergraduates in Biochemistry can significantly improve both formative and summative assessments (FAs and SAs) [1]. Our remediation strategy included a variety of methods such as mind maps, role play, viva, seminars, quizzes, small group discussions (SGD), weekly tests, quick revisions, assignments, grade incentives in FAs, summarization, open-book and closed-book tests, poster making, underlining and highlighting key points, leader-led group reading, independent study, and solving previous years’ papers. Feedback was collected from all participants to refine the remediation approach for future batches. The well-defined and interactive training methods greatly motivated the learners, who needed to pass the SA to advance to the next academic year. Students enrolled in remedial training showed improvement in both the third FA and SA results. The absence of a significant difference between the third FA and SA scores indicates that the improvement achieved through remediation was sustained over time. This underscores the effectiveness of the remedial methods. The relatively small class size facilitated effective interactions between mentors and students. A reward system, including individual mentorship from teachers, proved effective in boosting student motivation, compliance, and retention (96%) [11].

Common challenges affecting medical students’ performance include difficulty understanding the medium of instruction, unfamiliarity with medical terminology, dissatisfaction with career choice, perceived parental and peer pressure, excessive time spent on social media, influence of friends, anxiety, depression, lack of concentration, poor time management, early failures in class tests, and complete mental exhaustion [12–14].

Previous literature show various remedial strategies used to improve student performance. For example, Das et al., employed re-teaching and repeat examinations in pharmacology for 74 underperforming students. Four months of repeat classes on challenging chapters led to improved performance in repeat examinations compared to controls [15]. Similarly, Shankar et al., studied the impact of targeted remediation on 75 students who failed their first FA in anatomy. The study evaluated the effectiveness of a questionnaire-guided, three-month remedial program, which resulted in an increased pass percentage in the second FA for these students [16]. Mysorekar et al., employed counselling and training in stress-coping strategies as a remedial method for 83 underperforming students in pathology. The study revealed significant improvements post-program in student study behaviour, confidence, and self-esteem [13].

While previous studies on remediation for medical students exist, many have not standardized the strategies or adequately explained the active components of the remedial measures. These studies often focused on remediation of individual skills or student performance in exams rather than identifying the ideal teaching methods to enhance skills, critical thinking, and core learning abilities [17].

Compared to previous studies, our participants engaged actively with predefined remedial methods over six months. To accommodate student interests, less popular methods were used less frequently. We employed diverse learning methods to cater to individual student needs, often incorporating visual or interactive teaching-learning tools. We aimed to instil a sense of ownership in students, encouraging them to take responsibility for their learning. This approach aligns with findings from another study. In their article, Toit-Brits emphasized the importance of nurturing a sense of belonging in learning methods to encourage students to take ownership of their education. Students in remediation programs often experience a strong sense of non-belonging in group activities; addressing this through mentor support is crucial for their academic success [18].

To assess the effectiveness of these remedial resources in overcoming obstacles and achieving academic success, the frequency distribution of individual domains was analyzed. The perceived benefits of various remedial approaches were collected through a closed-ended questionnaire. Most responses indicated that mind maps, role play, viva, quizzes, weekly tests, quick revisions, open-book tests, and grade incentives in FAs were beneficial for academic improvement. The odds of using grade incentives in FAs as a remediation tool were six times higher (*p* < 0.05) compared to other teaching-learning strategies. This method remained an independent variable in determining academic success in students.

Timely feedback was provided to remedial students regarding their performance, likely enhancing their involvement and success in the learning process. Previous studies on remedial training have shown similar encouraging results [6, 10, 19], and our study aligns with these findings. While such interventions are beneficial for student progress, they also present several challenges. Many students do not take remedial measures seriously unless they are associated with some form of credit for their FAs. To address this, our study incorporated grade incentives as a key component of the remediation training. This approach led to improved student participation in all remedial modules, contributing to better grades in both FAs and SAs.

All facilitators initially underwent training to effectively deliver the structured remedial program. Motivated and well-trained mentors played a crucial role in helping students achieve their academic goals through successful implementation of the remedial training.

Initially, underperforming students often felt a sense of ‘non-belonging’ in these remedial classes. In our study, students preferred to refer to these sessions as ‘extra classes’ rather than ‘remedial classes.’ An orientation session with students and mentors was arranged to clarify the purpose and benefits of these classes. Once students recognized that these training methods were opportunities to enhance their learning skills, the stigma associated with remediation diminished. The remedial classes helped students accept feedback, acknowledge their deficiencies, and actively engage in various activities to bridge gaps in their learning abilities.

Our study implies the necessity of providing effective support to underperforming students who struggle to meet performance benchmarks. Remediation proves most successful when conducted in small groups and when the emphasis is placed on enhancing student skills rather than merely focusing on exam performance. Also, our study strongly advocates for the long-term monitoring of remedial students throughout their medical education to anticipate and prevent future academic challenges.

In future research, expanding the participant pool to include students scoring between 40 and 50% is critical. Multiple groups based on various score ranges (e.g., below 25%, 25–40%, 40–50%) can be formed, allowing for more tailored remediation strategies. This will facilitate better understanding of student needs, as different score groups may require distinct activities. Additionally, effective activities from the current study, such as grade incentives, should be retained and refined for larger groups. Reducing the number of activities per group while increasing their effectiveness will allow for better comparison and identification of the most beneficial interventions. This approach will enable targeted remediation and help determine which activities best improve academic performance and competency.

### Limitations

The study has several limitations. Firstly, the remediation was confined to the subject of biochemistry and was conducted only among first-year students at a single institution, limiting the generalizability of the results. The research could not be randomized due to ethical concerns. Additionally, sample size estimation was not feasible at the study’s onset due to its design. To mitigate response bias, the questionnaire was administered after the third FA results were released. However, the program’s highly interactive nature may have introduced a socially desirable response bias. Students scoring between 40 and 50% were not included as it would have been challenging to provide individualized attention to a larger group. Despite mentors being trained on the teaching methods prior to the study, minor variations in how each mentor conducted their classes might have occurred.

### Recommendations for implementing remediation in medical curriculum

Faculty training is essential for successful remediation. Training should include creative methods such as high-quality feedback, adopting a supportive attitude, recognizing and acknowledging student efforts, and offering timely rewards to enhance awareness of the psychosocial needs of underachieving students. Attaching some form of credit to formative assessments (FAs) can increase student engagement and attendance. The focus should be on skill improvement rather than just determining pass or fail status. Successful implementation of remediation requires strong institutional commitment to support all related activities. Institutions must back the resources and efforts needed for effective remediation programs.

## Conclusions

In conclusion, this study demonstrates that early identification of underperforming students in the medical curriculum and providing them with tailored remediation can significantly enhance their exam performance. Targeted remediation in biochemistry has proven particularly effective in improving students’ study skills, exam-related abilities, time management, and reducing negative perceptions of the subject. From the students’ perspective, grade incentives emerged as one of the most effective methods for improving their learning approach. Additional training tools, such as mind maps, quizzes, quick revisions, and assignments, also contributed to their progress. Given that this study was conducted at a single institution, we recommend extending this research to other universities to promote a student-centered learning approach and ensure the generalizability of these findings.

## Electronic Supplementary Material

Below is the link to the electronic supplementary material.

Supplementary Material 1

## Data Availability

Questionnaire used for the purpose of this study has been uploaded as a supplementary material. It is not added to the main manuscript.

## Abbreviations

CBME: Competency-Based Medical Education
FA: Formative assessment
OSCE: Observed structured clinical examination
SA: Summative assessment
SGD: Small group discussion
SSP: Short-integrated study skill program
P: Participants
