# Supplementary material for: Bridging knowledge gaps: impact of remedial classes on first-year medical students in biochemistry – a cross-sectional study
Source: BMC Med Educ. 2024 Nov 26;24:1375. doi: 10.1186/s12909-024-06243-y (PMC11590535; doi:10.1186/s12909-024-06243-y)
Supplement: Supplementary file 1 — Supplementary Material 1 [file 12909_2024_6243_MOESM1_ESM.pdf]

Annexure:1 Various remedial training methods employed in the study

Training modules	Purpose in remediation	~~Application~~ Implementation
Role play	• Apply theoretical knowledge to practical scenarios.<br>• Enhance memory retention through practical experiences.	Students were assigned with clinical case scenarios important for exam to enact in a group of five.
Mind map	• Organize complex information visually.<br>• Aid in understanding relationships between concepts.	Students were asked to make and present visually appealing posters of important clinical cases.
Seminar	• Reinforce understanding through repetition.<br>• Provide opportunities for deeper exploration and discussion.	Students were given important 3/5-mark topics for a quick 10-minute presentation before the start of the remedial class.
Quiz	• Assess understanding and identify areas needing improvement.<br>• Provide immediate feedback on comprehension.<br>• Encourage active participation and engagement.	A preliminary written quiz round was conducted for the entire batch. A 3-hour grand finale session included multiple rounds covering all competencies in Biochemistry. After each quiz round, there was an audience round which primarily targeted the remedial students.
• Leader led group learning<br>• Independent study	• Promote collaboration and peer support.<br>• Allow for targeted support and feedback from both peers and instructors.<br>• Address individual needs and pace of learning.<br>• Foster a sense of responsibility and autonomy in learning.	Interested non-remedial students from the batch were selected to lead groups of four underperforming students. These group leaders monitored their respective groups to ensure compliance with the remedial measures. In the process, the contributions of the non-remedial students were acknowledged, ensuring they benefited equally.
Weekly test	• Provide regular feedback on progress.<br>• Reinforce learning and retention of concepts.<br>• Identify areas of weakness for targeted improvement.<br>• Help track individual student performance over time.	Underperformers had to undergo weekly test and were given feedbacks on their progress. Mentors counseled each student one on one and motivated them for better performance in the future.

Quick revision	• Reinforces key concepts quickly.<br>• Provides focused review of challenging topics.<br>• Prepares students for assessments and exams efficiently.	Students had tests after the practical hours. Before the commencement of test students were asked to highlight and underline the answers for the important topics.
Summarization	• Ensure clarity and understanding of fundamental concepts.<br>• Provide opportunities for active engagement and note-taking.<br>• Reinforce learning through repetition and summarization.	Before beginning the didactic lectures, remedial students were informed that one of them would be called upon at the end of the class to summarize the topic covered. This encouraged them to take notes and remain attentive during the class.
Assignments	• Provide opportunities for self-assessment and reflection.<br>• Offer individualized practice to address specific areas of weakness.	Students were asked to write long answer essays for in-depth assignments after each topic. Brief assignments were given quick short answer essays for core "Must Know" topics.
Grade incentives in FAs	• Encourage active engagement and participation in learning.<br>• Improve student attendance in all activities.	Performance and compliance of students towards the remedial methods was assessed and scored out of 6 and this mark was added to the FA to boost the student's performance. This strategy helped the students comply well with all other remediations.
